# A Memoir on the Employment of Terebinthinous Remedies in Disease

**Published:** 1821-08

**Authors:** James Copland

**Affiliations:** Member of the Royal College of Physicians, London.


### *A Memoir on the Employment of Terebinthinous Remedies in Disease.*

By James Copland, M.D. Member of the Royal College of Physicians, London.

TO inquire into the operations of old and familiar remedies, which the experience of ages has proved to be of service in disease, notwithstanding their liability to injudicious prescription and consequent disrepute; to examine into their mode of effecting salutary changes in the system, and to attempt to give precision to our ideas respecting that influence in disease; to show how far they are really deserving of their reputation, what share of benefit results from their individual efficacy, and what to nature herself, or how great may be their separate influence in rousing and directing the vital energy of the system,—would be a work of no small utility, both as tending to increase our stock of useful knowledge, and in banishing from the field the empirical exhibition of remedies, amongst even the legitimate members of our profession. To those who are entering upon the study of the healing art, it might be expected to prove of considerable advantage, as it would tend to arrest their attention to the philosophical consideration of the process by which their effects are produced, and to connect the remedy with the pathology of the disease in its various stages, and in its more complicated connexions. It may be urged that such a process would require a degree of knowledge greater than we at present possess, respecting not only the effects of individual remedies upon the general systems of the body and its separate textures, but also of the physiological pathology of disease, or those early aberrations from an healthy state of an organ, which mark the commencement and first stages of maladies. I am, however, of opinion, that the state of our knowledge in the fundamental and collateral sciences of medicine is at present sufficiently advanced to furnish us with materials from which to draw incomparably more correct and more important conclusions than those with which we have been hitherto contented. The difficulties with which such an undertaking would be surrounded, are certainly not sufficient to discourage many members of the profession from its execution, by which an important desideratim in our otherwise excellent pharmacological and therapeutical works would be supplied.

Having premised these observations with the view of directing the attention of the profession to a closer examination into the process by which remedies operate their effects upon the animal economy, I shall now proceed to make a few practical remarks on the employment of the oleum terebinthinæ as a remedy in various diseases, in continuation of those offered to the public in the preceding Number of this Journal. Although my observations are apparently confined to the essential oil, they may be considered as applicable to all the varieties of turpentine, which chiefly owe their virtues to the quantity of the oil that they contain.

The turpentines were extensively employed in medicine by the ancients, and were probably in use long before the days of Dioscorides* and Pliny, who appear to have recommended them in many diseases, besides those in which they are prescribed by the moderns.

It is most probable, from the eulogy which Pliny† pronounces on the Pistacia terebinthus and lentiscus, and from their former abundance in the South of Europe, in the North of Africa, and in Asia Minor, that the turpentine yielded by these species was in most general use, not only amongst the Greeks, but also among the Egyptians and Romans. That they did not confine themselves, however, to the production of any particular species, appears even from the earlier writings of Celsus,‡ who, although he makes no mention of this substance, yet frequently recommends various parts of the different species from which it is obtained, as a remedy for similar diseases with those in which they are recommended in the more modern writings of Dioscorides and Pliny. Thus, he frequently prescribes "Flos, nuclei ex pinu silvestri, et pineus cortex;" (see pp. 143 et 248:) and also adds, "Nuclei pinei stomacho idonei, urinam movent, semen contrahere videntur; non inflant, cum melle edendi," &c. (p. 68 *et passim.*) Other instances could be given, proving that the bark, leaves, &c. of the various pines were used by the Roman physician, both as an internal remedy and as an external application; but no one can hesitate in assigning the benefit which experience had shown to be derived from them to the essential oil they contained.

The most decided proofs of the general admission of all the varieties of the turpentines into the materia medica of the ancients, may be gathered from the writings of Dioscorides,

* Dioscor. lib. i. cap. 91, p. 50.

† Plinii *Historia*, cap. xxiv. sect. vi.

‡ Celsus *de Med.* passim.

Pliny, and Aretæus. The first-named author, in his second book, classifies the turpentines into moist and dry. Pliny adopts the same arrangement; and both enumerate very fully the different species from which each variety is obtained. "Summæ species duæ, sicca, et liquida. Sicca é pinu et picea fit: liquida è terebintho, larice, lentisco, cupresso." (Cap. xxiv. sect. vi.) This enumeration accords very nearly with that given by Dioscorides; as do also his remarks upon the manner of employing them internally and externally in the diseases in which they were prescribed.

Aretæus* has been still more particular in his recommendation of this substance. He gave it internally, and frequently combined with nitre and rue, making honey the vehicle, in lethargy,† apoplexy,‡ melancholia,§ and in pleuritis.‖ The liquid turpentine was prescribed in pulmonary affections,¶ given in the combination just mentioned, but resorted to after venesection. It was also frequently recommended by him, in those diseases, as an outward application at the same time. He prescribed it also in enemas, in cephalea** and volvulus,†† in conjunction with cummin and rue; in phrenitis‡‡ and tetanus§§ as an external remedy; and, in the latter disease, it formed the principal ingredient in the cataplasms which were ordered to the injured part, upon the supervention of the tetanic symptoms.

Since the days of Aretæus, the turpentines continued to keep their place in practice. Prosper Alpinus‖‖ enumerates them amongst the individual remedies composing the Theriaca of the Egyptians, during the period of his residence in that country; and it is probable that they had retained their station for many ages. That they were a constituent in the polypharmacy of the middle ages, and entered largely as an ingredient into the farraginous preparations, which were both exhibited internally and applied externally in those times, may be easily conceived, from the later, but yet extravagant, praises of Fernelius.— "Terebinthina calefacit, mollit, discutit, terget, expurgat: viscerum omnium, maximeque renum, obstructiones tollit, et angustos meatus aperit, urinam ciet, putredinem cohibet."¶¶

As the chemical remedies and the chemical doctrines of disease became disseminated through Europe, the province of the terebinthinous class of medicines became invaded, their boundaries more confined, and, with many other celebrated galeni-

---

* Aret. cura Boerh. p. 76—126, passim.

† P. 79, B. ‖ P. 91, B. †† P. 106, D.

‡ P. 82, A. ¶ P. 94, B. ‡‡ P. 76, C.

§ P. 126, B. ** P. 116, B. §§ P. 86, B.

‖‖ *De Medicina Egypt*, lib. iv. p. 305.

¶¶ Fernelii *Opera*, vol. i. p. 444.

cals, deprived of their due consideration. The diseases of the urinary organs were left, however, in their almost undisturbed possession; so that, up to the present day, they have always attached to themselves a portion of attention in the alleviation or cure of disease.

PART I.

Reasoning from the apparent effects of the oleum terebinthinæ upon the animal economy, and from what I conceived to be the correct pathology of various diseases, I considered it a medicine of more general application than that hitherto assigned it. Although my experience of its efficacy has been rather limited in regard to some of the diseases in which I have ventured to recommend its use, yet I consider the success sufficiently great to entitle it to more general adoption. Its disagreeable odour, and its liability to produce offensive eructations and even nausea, but rarely actual retching, may have tended to prevent its more general introduction into practice. Those disadvantages, however, are easily obviated, and are certainly insufficient reasons, even were they just, against resorting to its aid in several diseases in which I am certain no other remedy can be found to produce more decidedly beneficial effects, when exhibited to fulfil correctly-formed indications, and with the view of accomplishing these only.

However weighty this objection may prove in our practice among the fashionable and fastidious,—those whose digestive organs possess no great share of energy,—it should have little influence in preventing its employment in dangerous ailments; and surely never, under almost any circumstance, amongst the labouring classes and the objects of the numerous medical institutions in the metropolis and large towns throughout the kingdom. This objection, which I have heard urged against this substance, and which on that account I now notice, may be frustrated, in a great measure, by a judicious combination or method of exhibition. Dr. Paris, in his very excellent work on Pharmacology, furnishes the hint for one mode of getting rid of this source of annoyance; which is, by the addition of a few drops of some fragrant volatile oil, as that of lemons. This is in some respects similar to that which I have been long in the habit of using,—namely, giving with it some aromatic tincture or warm spice. In an intertropical climate, where I have found it necessary to prescribe it to individuals of weak digestion, and accustomed to large quantities of the hot spices of the country, the oil seldom failed of producing considerable nausea, if exhibited in a great dose, unless accompanied by a very large portion of such a corrigent.

*Modes of Exhibition.*

I. *In regard to the quantity.*—The quantity of this remedy prescribed in a single dose ought to vary with the indications that lead to its adoption. 1st. When it is desirable that it should operate through the medium of the circulation, and be conveyed to secerning vessels and capillaries, it ought to be given in a dose of six or eight drops to one drachm, according to the frequency of its repetition. This may be called the *small* dose of the medicine. If exhibited in this quantity, it generally exerts its influence upon the kidneys, and hence proves diuretic. This virtue it, however, possesses more from the direct stimulus which it affords to that organ, than from any influence it exerts upon the absorbent system. In addition, however, to its effects upon the kidneys, it produces a very important one upon the exhalants and terminal vessels, when given in this proportion. It constringes their diameters by increasing their tone; and by these means proves beneficial in atonic hemorrhagies and dropsies.

2d. When given in a quantity which I consider a *medium* dose, that is, from one to two drachms, its effects upon the system are more general. But, then, these effects are entirely controlled by the duration of the intervals between the repetition of the remedy. If given in a single dose only, or if repeated after a longer period than six hours, it generally is entirely or in part absorbed, and may affect the urinary organs to excess. In addition to its action in this manner, it frequently gives a salutary impetus to the digestive tube; and if the mucous coat, with its absorbing and secreting apparatus, is in a state of chronic disorder, a salutary change is frequently produced. When exhibited at intervals of less duration than six or eight hours, the latter effects are more manifest; whilst its operation through the medium of the circulation may be more or less marked, according as it has been given at longer or shorter periods, combined with other remedies, or as it may be owing to the circumstances of the individual. Instances of various diseases are of frequent occurrence, wherein it is desirable to produce this extended operation of the remedy,—cases requiring its more general effects upon the system, as well as its immediate influence upon the intestinal canal. This mode of exhibition may be resorted to in some varieties of epilepsy, in chorea, chronic hydrocephalus, &c.

3dly. The third, or *maximum*, quantity which may be taken of this substance, and which may be termed a full dose, is from four drachms to two ounces, according to the circumstances of the case and of the individual. If taken in this quantity, it generally operates as a cathartic; but that effect cannot be depended upon, it may produce an opposite one; and in that case it will be absorbed in excess, and may produce noxious effects. Having experienced its influence under such circumstances, I have since either given it along with another cathartic, or exhibited one soon afterwards, whenever it has been slow of producing a purgative influence. The advantages arising from employing the oleum terebinthinæ with this intention, are chiefly from its concentrating the vital energies towards the organs of digestion and chylifaction, and by those means effecting a more complete and permanent revulsion of the impetus of the circulation from the great nervous centres. Its effects upon the internal membrane of those organs, and the vessels opening upon its surface, are more efficient; and it tends to remove the viscid mucus and disordered secretions often obstructing their functions.

When exhibited in this quantity, experience has now proved its certain and almost specific influence as a vermifuge.

II. *Respecting the method of exhibiting and combining this remedy, so as to render it more agreeable, and at the same time to assist its effects in the different diseases in which it may be prescribed.*—This is a subject of importance as connected with the exhibition of this remedy, and indeed not of this alone, for it is an important consideration in the internal use of most of our active medicines. Dr. Cullen* found the unpleasant sensations consequent to the ingestion of the terebinthinous remedies in any large dose, a considerable bar against their employment among delicate females, in several diseases in which he considered them of important service. Had he, however, prescribed them, even in the manner recommended by Aretæus,†—that is, combining them with some other aromatic stimulant, and making honey the vehicle,—his objection would have been in a great measure removed.

1st. It may be prescribed in either of the already-mentioned proportions, floating upon the surface of a draught composed of some agreeable distilled water, with the addition of a drop or two of some fragrant oil and a little warm aromatic tincture. The addition of two or more aromatics to this remedy will prevent it from producing any degree of nausea, or pain in the stomach, which its uncombined exhibition sometimes occasions. The propriety of employing hot aromatics in this manner has been judiciously noticed by Dr. Paris;‡ and I can fully confirm his observations, not only in this particular remedy, but also in

* *Materia Medica,* art. Terebinthinæ.

† Aret. *in loc. ant. cit.*

‡ *Pharmacologia,* p. 104 et passim.

many others which I have found it requisite to prescribe in irritable states of the stomach, surpassing what is usually met with in temperate climates.

Thus, in the intermittents which prevail during the rainy seasons in hot climates, the state of the stomach is usually so irritable that it is with difficulty the smallest doses of the bark of cinchona are retained, when exhibited uncombined. If given, however, combined with aromatics to a degree proportioned to the habits of the individual regarding the use of those condiments, the dose of this admirable remedy may be carried to a surprising length, without producing effects in the least degree unpleasant. In this manner I have frequently exhibited half-an-ounce of cinchona at one dose, with the unvarying effect of putting a stop to the disease. The corrigents which I have thus been in the habit of prescribing most frequently, are the carb. of ammon. camphor, some of the hot aromatic spices, tinctures, and essential oils. The capsicum annuum I have found, both from personal feeling and from experience, with others, to possess the most pleasant and most permanent effects in correcting and in preventing the disagreeable sensations arising from other remedies.

2d. Another mode of administering this remedy, independently of the quantity, is by suspending it in the vehicle by means of some mucilage. When given in this form, the necessity of conjoining it with another essential oil, in order to subdue its odour and correct its effects upon the stomach, is still required: the compound spirit of ether may be also employed with that intention; the tincture of capsici, or any other warm aromatic spirit or tincture, may be also added. This mode of exhibition I have never found to produce nausea; but, even when given in a large dose, its absorption is, in my opinion, thereby promoted.

3d. A third form under which it may be taken internally, and one in which it was generally exhibited by the ancients, is that of a linctus made with honey, or with any rich aromatic syrup. This mode may be chosen when it is desirable to give it in small doses: the necessity, however, of prescribing in the same recipe some fragrant and aromatic preparation must be sufficiently obvious when we direct the remedy in this form.

I have been of late frequently in the habit of prescribing this medicine for children of almost all ages, and in different proportions, but generally in the maximum dose; and I do not recollect an instance wherein it has not been taken by the child, or wherein it was afterwards rejected.

The lively author of the article upon Cookery, in the 69th Number of the Edinburgh Review, (p. 45,) ingeniously considers that the flavour of any substance arises from the odoriferous particles escaping during the process of mastication, and while it is held in the mouth, through the posterior nares, and there, at the same time, affecting the organs of smell. In pursuance of this doctrine, and for the benefit of those who are doomed to copious draughts of nauseous remedies, I would recommend that the olfactory nerves, whether anterior or posterior, should be secured as far as possible against the invasion of the disagreeable odour; both while it is external to those watchful sentinels, or even after it has entered the portals which they so faithfully guard. The manner in which this may be effected requires not my formal prescription.

### PART II.

### *Observations respecting the particular diseases in which it may be employed.*

*Chronic Rheumatism.*—The terebinthinous remedies have been long in use in several varieties of this disease, as in lumbago, sciatica, &c. and experience has long demonstrated their essential service. Drs. Pitcairn and Cheyne were the first to recommend the essential oil in this affection; afterwards Dr. Home and Dr. Cullen* employed it frequently. Home† gave it in the form of a linctus, consisting of two drachms of the oil and one ounce of honey; and of this a tea-spoonful was taken twice or thrice in the twenty-four hours. This was the same formula as that recommended by Cheyne; and, from the cases related by Home, it appeared to have generally cured the disease.

In more modern times, it was highly recommended in combination with the preparations of cinchona in this disease; but, as cases were of every-day occurrence in which cinchona alone produced unpleasant effects in the stomach, the addition of the oil only did not remove that objection, and it appears never to have come into very general use. When this remedy is employed in chronic rheumatism, it may be taken either in the small or medium dose, and may be combined with any of the preparations of cinchona, or with the senega, &c. triturated with mucilage into the form of a draught; to which tinct. capsici, or tinct. cardam., or spirit. armorac. comp., with a drop of some essential oil, ought to be added, in conformity with the observations already offered. The capsicum annuum‡ is, in my opinion, preferable to every other, both as an adjuvant and as a corrigent to other remedies in this disease;

* *Mat. Med.* loc. ant. cit.

† *Clinical Experiments*, p. 246 et seq.

‡ I shall give, in a following Number of this Journal, some observations on the medicinal qualities of this substance.

for I have ascertained that, if given in considerable quantities, in the form of pills, it will of itself remove this disease as soon as any other remedy.

*In Sciatica*, I have found the following linctus always efficacious:—℞. Mellis optimi, uncias duas; olei terebinthinæ, tincturæ guaiaci ammoniatæ āā, drachmas duas; olei caryophil. et olei limonis, āā M iij. Misce ut fiat linctus: cochleare unum minimum bis terve de die sumendum.—The operation of the turpentines should be watched, lest they affect the urinary organs. I am much inclined to suppose that the use of diluents, if resorted to at the same time, will tend to determine their action towards the kidneys and urinary passages.

*In Lumbago*, I have never employed this medicine. Dr. Darwin* found it of service in this variety of rheumatism, and gave it in the dose of one drachm twice a-day.

Respecting its mode of operation in removing this complaint, I consider it to act like most stimulants possessing a permanent character. Its action in this disease cannot, however, be readily explained, while the pathology of the disorder remains unestablished. The prevailing opinion is, that this affection consists in a slow inflammatory action, or inflammation of some sort, existing in the fibrous textures of the body, or even sometimes in serous membranes; but it is now considered doubtful whether it actually does arise in muscular fibres. To this pathology has been urged the objection, whence comes it that rheumatic inflammation, if it be actually such, never terminates like inflammation in other textures? Because it is seated in *unyielding* and *thin* textures, can be the reply: the former quality preventing that relaxed state of the capillaries necessary to suppuration; the latter, as it allows its immediate connexion to sound parts, along one of its surfaces at least; and from no part, in consequence of this character, being removed to a sufficient distance from the healthy interior and exterior tissues to permit of its sphacelation, before the neighbouring textures participate in the disease.

But it may be again inquired, if it be granted to be an inflammatory or any analogous state of the capillaries, existing in a peculiar species of texture, whence arises its sudden invasion, and rapid termination, from causes which operate directly upon the nervous sensibility? To this I would answer, that impressions made primarily upon the nerves of a part may influence, for longer or shorter periods, the state of the lymphatic and capillary arteries.† That such influence may be equally exerted, whether it takes place through the medium of the

* *Zoonomia*, vol. ii. sect. iii. chap. 2, sect. 6.

† By lymphatic arteries, I mean the colourless vessels, not admitting red blood.

system, or from the local influence of the causes. The mode of either being brought about, I must reserve to another opportunity to show: let it suffice at present to say, that rheumatism, and even gout, most probably arises from constitutional or local and fortuitous causes operating changes upon the nervous constitution of a part; and that the primary affection in the nerves cannot long exist, without inducing changes in the state of the capillary circulation of a proportionate degree of intensity.

Such, then, being the view I entertain of this disease, I consider that the substance now under consideration operates in removing it, primarily, from the stimulant effects it produces upon the digestive organs, and by nervous communication upon the heart and other textures; and afterwards, when it becomes absorbed into the current of the circulation, it produces those effects in a more signal manner, by being conveyed to the very seat of ailment, and by stimulating the nervous fibrillæ distributed upon the affected capillary vessels, restores them to their natural tone and energy.

*In the hemorrhagiæ*, I consider it a remedy of almost immediate efficacy in many of the affections belonging to this class. It requires, however, great discrimination on the part of the physician to determine the precise circumstances of the patient, under which it shall prove of decided advantage; the quantity of the remedy requisite to produce the desired effect; and when to stop in its exhibition; for, if given in very small doses, so as to become absorbed into the circulation, without producing much previous increase in the action of the heart, it will generally prove of essential service: whereas, if the dose exceed even a few drops, if it be repeated too frequently, or if its use be too long continued, an increase of the disease may be the consequence. The effects, however, depend entirely upon the state of the habit and diathesis of the individual. A phlogistic temperament, even although the debility may be considerable, ought to forbid its exhibition. The best general indications for the internal employment of this remedy in hemorrhagic diseases, and those by which I have been guided, are the absence of plethora and the phlogistic diathesis; when there has been a previous great loss of blood; when the pulse is soft, weak, and easily compressed, indicating a want of tone in the arterial system. In a word, it can be confidently resorted to in all hemorrhages possessed of a truly passive character.

In many cases of hemorrhage displaying what has been commonly called the hemorrhagic effort, as preceded and marked by a sense of titulation, heat, &c. this medicine, if properly watched, is not contra-indicated; for, if it be given in such a quantity as to produce a considerable concentration of the vital energy towards the digestive canal, a revulsion may be effected from the part threatened, and thus, by the exhibition of the maximum dose, the occurrence of the disease prevented, at least for that period; and afterwards, by the cautious use of small doses of the medicine, so as to convey it into the mass of fluids, a degree of tonic and astringent effect will be imparted from it to the capillary vessels and arteries, sufficiently great, and indeed greater, than is barely requisite to withstand the impetus with which the blood may be propelled along their canals, and to preserve their integrity against the impulse given to their contents by the increase that takes place in the strength of the heart's contractions.

In the *Atonic Epistaxis* of children, I have more than once found it of service; and I consider that it may be resorted to in many cases of *Hæmoptysis*, when that disease is accompanied with great debility, with considerable, although seldom with permanent, advantages. My friend Dr. Clutterbuck lately found it of service in such a case; and I am sure there are many cases, usually ranked under this denomination of disease, wherein the deranged structure only exists in the mucous membrane of the bronchia and air-cells, or amounts only to a relaxation of the capillary vessels on those surfaces, which would be considerably benefitted by the exhibition of this and other terebinthinous remedies, and would thus justify the employment of those medicines in pectoral complaints by the ancients.

When, however, there is reason to suppose that this disease arises from ulceration in the substance of the lungs, injuring the integrity of a considerable blood-vessel, even though much relaxation of the nervous and arterial systems were present at the same time, the use of the oleum terebinthinæ could be but of doubtful advantage; unless we suppose that, if conveyed through the current of the circulation, it may produce a beneficial effect upon the ulcerated surface, in the same manner as a diluted application of it frequently does in external ulceration.

In *Hæmorrhois*, especially when seated high in the rectum, as they frequently are in those who have suffered from repeated and continued attacks of dysentery in warm climates, I have sometimes found the turpentines of partial advantage, and have employed them as an adjuvant to other remedies. When this disease arises in individuals from the causes just alluded to, there is generally impeded or obstructed circulation in the liver, or, then, some degree of ulceration and stricture in the rectum, from its being frequently the seat of disease; under either of which circumstances, although the oleum terebinthinæ, and substances that yield it, may arrest the bleeding, the constitutional disturbance will not be diminished.

In *Menorrhagia*, I have no experience of its effects; but I strongly entertain the opinion that the oleum terebinthinæ is calculated to be of the most essential benefit in puerperal menorrhagia or flooding, if given in a dose of an ounce or upwards. If exhibited in this quantity, I have no doubts of its influence in promoting the contractions of the uterus; and by these means producing the constriction and varied inflections of the vessels, affords a mechanical, but at the same time a natural, obstruction to the flow of blood, and soon terminates the mischief. Against this most alarming of all natural hemorrhages, I also consider an enema of at least two ounces of this substance ought to be immediately administered. I recommend this, with the utmost confidence of success, to those whose practice affords them opportunities of putting it to the test of experience.

In *chronic Dysentery*, unconnected with disordered liver, and in those cases in which the disease is marked with a relaxation of the capillaries and mucous follicles, I have in several cases witnessed its decided utility. If it be had recourse to as an enema in this disorder, it should not be employed while the disease partakes of any of the acute symptoms; and, when resorted to, it ought to be in small quantity, as it will otherwise produce a return of the tenesmus and other distressing symptoms.

When given in the purely chronic form of this disease, the medium quantity may be chosen, and exhibited in a combination which the circumstances of the case will suggest. I have preferred it suspended in a mucilage from the compound powder of tragacanth, with any of the astringent and tonic extracts or tinctures, and the aromatic corrigents. It may also in this manner be given along with rhubarb and ipecacuanha.

In the *chronic Diarrhœa* of children, in which there is reason to suspect slow inflammatory action to exist in the mucous coat of the intestines and in the follicular glands in that situation, few remedies, (if it is suitably combined, and given in a properly-digested plan, according to the circumstances of the case,) a e likely to be of more permanent service.

The plan I have generally pursued in those cases, has been to prescribe a powder at bed-time, composed of the hydrarg. submur., pulv. ipecac. comp., pulv. rhei. or the pulv. tragacanth com., in proportions according to circumstances and the age of the child; and a draught in the morning, of the oleum terebinthinæ enveloped in mucilage, with the requisite corrigents. A medium dose in this affection has been the quantity usually prescribed.

In *Apoplexia.*—I have never prescribed it in this disease, but I think it may be employed with advantage in a maximum dose, from the revulsion it would produce of the circulation from the head. I am also of opinion that, in many varieties of *paralysis*, it would be calculated to prove of service, from its restraining the disposition to congestion and effusion upon the great nervous centres, and by rousing the ganglian nerves to increased energy. In order to produce this effect, it should be given in small and repeated doses, to admit of its absorption. It may be also exhibited as a purgative, in cases requiring the active operation of such medicines.

In *Epilepsia.*—Dr. Percival was the first to recommend the oil of turpentine in this disease. Drs. Latham* and Lithgow† afterwards employed it with success in the same affection. From the circumstances of the cases detailed by these physicians occurring in females, and from its tending to promote the regularity of the menstrual flux, it has been argued that the benefit derived from its exhibition arises from its emmenagogue operation. It has been given, however, in cases wherein such a mode of operation was impossible, and with the most decided advantage. From what I have seen of its exhibition in several cases, both in my own practice and in that of others, I certainly consider that, in the majority of epileptic cases, whether occurring in the male or female, in the child or in the adult, no medicine can be administered with more certain prospects of success, when judiciously given or sufficiently persevered in. The manner in which I have prescribed it, is in small doses in some tonic or antispasmodic mixture, at the same time attending to the state of the bowels. Exhibited in this form, it becomes absorbed, and tends to promote a regular distribution of the nervous influence throughout the various organs of the body, and to support an equable degree of tone in the capillary circulation in the great nervous centres. If, however, this mode of exhibiting the remedy affect the urinary passages, its use may be intermitted for some time; as such an occurrence shows its presence in the circulation in a quantity sufficient to produce some degree of stimulus upon the terminal vessels.‡

The good effects of the oil and of the turpentines, when employed in this manner, arise, in my opinion, (which seems to be confirmed by the experiment related in the preceding Number,) from this essential oil being conveyed into the circulating mass, and there producing a more general and more decided effect

* *Edin. Med. Jour.* vol. ix. p. 493. † *Loc. cit.* vol. xi. p. 300.

‡ By *terminal* vessels, I mean the extreme capillaries, whether running into veins or into those conveying colourless lymph, or terminating in any of the textures, whether into their substance, upon their surfaces, or into any other series of vessels, &c. which they may possess.

than its local influence upon the digestive organs could have occasioned. I consider this substance as one of the few vegetable products that are not decomposed, nor materially altered, by the process of digestion ; and that, like the nitrate of silver, the sulphate and oxide of zinc, and the solution of arsenic, whose operation in the cure of this disease is so remarkable, it is not resolved into simpler elements during that operation. I may here add, that those mineral substances just enumerated can be proved to exert their influence in this and in other diseases, from their presence in the circulation ; but, whether they act by exciting the vessels in the quarter supposed to be the seat of the affection to a more regular and continued state of tone and energy, or whether such regular exertion of vascular action, kept up in the vessels ramified in the nervous centres, promotes the production and exaltation of nervous influence, which, as soon as produced, is distributed through the various channels, and ultimately influence the vessels that gave it origin, the reciprocal effects thus moving in a continued and unvarying chain, is a matter of difficulty to determine. The effects may be produced in either of these modes, but most likely by them both conjointly ; that is, by the continued stimulus afforded the internal coats of the capillaries, and by the consequent state of the circulation in them generally, and, in those distributed to the nervous centres particularly, favouring the production of nervous influence, and its regular distribution throughout the system.

Having employed this remedy in the intervals in the manner just mentioned, care should be taken to ascertain the exact period in which the invasion of the paroxysm takes place ; and as, in most cases of the purely cerebral variety of the disease, the fit occurs during the night, a large dose of the oil, generally about an ounce, but more or less according to the circumstances of the case, with about half that quantity of the oleum ricini, ought to be taken so soon as the patient is in bed. By giving it when the patient has retired to rest, it is less apt to offend the stomach in irritable states of that organ. In many instances, this mode of proceeding will prevent the accession of a fit, even upon its first employment; it will at least, in most instances, defer the paroxysm. But, in more obstinate, and in even hereditary cases, by pursuing the disease in this manner, the epileptic seizures will at first become less severe ; their attacks shall be afterwards deferred ; and, by pushing the continued small doses of the medicine during the intervals, so far as the state of the urinary organs will permit, and by anticipating the paroxysm by a full dose of the oil, they may be expected gradually to disappear. When this end is attained, the medical treatment and regimen ought to be directed to preserve the nervous energy and tone of the system, and to obviate plethora. It will, however, generally be found in epileptic patients, that the treatment most conducive to effect the former shall also accomplish the latter.

In the epilepsy of children, which generally arises from the irritation of worms in the alimentary canal, or from a disordered state of its mucous membrane, this medicine will seldom fail in proving of immediate advantage. It acts more speedily than any other in removing the cause, and consequently, in such cases, the disease.

Before leaving the subject of epilepsy, I cannot help regretting our want of such an officinal remedy as the essential oil of valerian. It is an admirable remedy in both this disease and in hysteria, besides in many other complaints in which antispasmodics are indicated. It is to this oil that the virtues of all the preparations of valerian are owing, and it possesses the advantage over them of being infinitely more active and more easily and more pleasantly exhibited, especially to children, against whose diseases it is so frequently efficacious. It is highly esteemed in the North of Germany, and there supersedes every other preparation of valerian.

In *Chorea.*—I have prescribed the oleum terebinthinæ in two cases of this disease. In the one detailed in the January Number of the Medical Repository,* which alternated with rheumatism, and in which the disease affected the heart, and was ultimately fatal, it proved of no service; but it must be recollected that it was not resorted to until the very extensive derangement of structure, detected upon dissection, had proceeded to an irremediable heighth. The other case was speedily removed by this medicine, given generally in a full dose, and sometimes with a little of the oleum ricini to promote its cathartic effects.

In *Tetanus.*—I have no experience of its effects in this disease. In one very acute case of this frightful malady, that recently came under my observation, I intended to have given this substance a trial. It, however, terminated fatally before it was exhibited, in a more rapid manner than was expected. Dr. Phillips† relates a case of the idiopathic species of this disease, which was almost instantaneously cured by this remedy.

*Convulsio.*—I have in several instances witnessed the effects of this medicine in the convulsions of children, especially the sympathetic variety which originates in a disordered state of the alimentary canal. In such cases, I have given it so as to produce its cathartic operation, and always with the best suc-

* For 1821, p. 23.

† *Medico-Chirurg. Transactions*, vol. vi.

cess—the removal of the affection. In the convulsions proceeding from a primary disease in the brain, as they follow the course of such maladies, they will be considered in connexion.

In *Raphania*.—Three cases of this singular species of ailment have occurred to my observation in children. In one of nine years of age, which was particularly interesting on account of the nature of the muscular contractions, and indeed the whole phenomena accompanying the disease, but which my limits forbid me at present to detail, the oleum terebinthinæ was given, without producing any effect; but it must be mentioned, in justice to this substance, that every species of treatment equally failed. The arsenical solution, nitrate of silver, the preparations of zinc, and courses of cinchona and of antispasmodic remedies, also proved inefficacious. No appearance of irritation could be traced to the spine, to the brain, to the intestinal canal, nor to the gums. The child at last became suddenly exempt from seizure, six months after having made use of any remedy. In the two other cases, this medicine proved completely efficacious.

In *Colic*.—I have never ordered this substance in colic. Dr. Cullen* recommended it in the flatulent variety of this disease, as an enema. I consider this form of exhibition the most eligible, and the flatulent species the only kind of colic in the cure of which it can be admissible.

In *Diabetes*, it certainly deserves to be tried, not by itself only, but also in combination with other remedies, as cinchona, &c. Dr. Darwin† recommended it in this disease, but has not mentioned his experience of its effects.

*Hysteria* presents several varieties, in which the terebinthinates may be employed with advantage. In those forms of the disease which arise from debility induced by menorrhagia and fluor albus, or in those connected with a vitiated state of the stomach and intestines, they may be given in doses and in a manner suited to the circumstances of the case; and either in such a mode as to be absorbed into the circulation, or to evacuate fully the contents of the intestinal canal.

Of the employment of the oil of turpentine in *Hydrophobia*, I cannot speak from experience; nor am I acquainted with any trial having been made of its effects in this dreadful disease. It certainly deserves experiment.

In *Mania*.—I am unable at present to speak of its effects in this malady. I have, however, one case under treatment, in which I have prescribed it in a medium dose, so as to operate gently upon the bowels, and by these means to allow its partial

* *Materia Medica*, loc. ant. cit.

† Vol. ii. sect. vi. 2, 4.

absorption. Sufficient length of time has not yet elapsed to permit us to be acquainted with its effects.

In *Marasmus*, accompanied with a diseased state of the mucous membranes and the vessels and follicular apparatus opening upon their surface, I consider that the occasional employment of a medium dose, or even any other quantity of this medicine, will be found of considerable service. In a very few such cases in which I have prescribed it, I have found it advantageous.

In the *Hydropes*.—The oleum terebinthinæ and the terebinthinates have been long prescribed as a diuretic, especially during the two last centuries, by the German and Dutch physicians. Fonseca* gave the latter in barley-water, with this intention. Closius† exhibited them in the form of pills, combined with large doses of the sulphate of zinc. Such a formula he recommends for its diuretic properties. Werlhof and Stahl‡ extol also their effects in dropsy; and Holst§ and Pop|| praise them highly in such diseases, in Hufeland's Journal and Roeschlaub's Magazine: the latter writer considers them to be an excellent application to the roots of sickly plants.

Wilkes¶ says, "The etherial oil of turpentine is a most powerful diuretic." Hardinge prescribed it, with this intention, in combination with an infusion of juniper berries and nitre, and directed it to be administered as an enema. Bang added it to olive oil, and recommended it to be rubbed over the abdomen in ascites.

In *Anasarca* and *Hydrothorax*, arising chiefly from a relaxation of the lymphatic capillaries, and marked by a serous or leucophlegmatic character of countenance, I consider that the terebinthinous remedies may be employed with advantage. But in neither can they be admissible when the arterial action is in any degree of exaltation, as it always is, either generally or partially, in the forms of those diseases long known under the appellation of acute dropsies. In those species, also, of dropsical diseases where the energy of the nervous influence, or moving power, is insufficient to the mass of fluid to be held in motion, and congestion in the venous trunks of vessels become the consequence, either generally or partially according to the extent of the cause, the terebinthinates may be resorted to with advantage. The doctrine of congestion, as laid down and illustrated by Stahl,** has been in the present day called to the

* *Consultationes Medicæ*. Venet. 1620, passim.

† *Problemata aliquot Argent*. 1649.

‡ Stahl. *Theor. Medic. vera, Pathol. spec.* P. iii. p. 858, 862.

§ Hufeland's *Jour*. B. xx. 2 st. p. 147.

|| Roeschlaub's *Magazin*, B. i. 3 st. 10 n.

¶ *On the Dropsy*, p. 264.

** *Theor. Medica vera*, passim.

explanation of the phenomena of diseases which cannot be so distinctly derived from such a source ; but, in whatever disease such a state of vessels may be proved to have existed, it surely forms only an intermediate link in the chain of causation ; an earlier one may be found in the nervous system. In the dropsies which derive their origin from any considerable structural derangement existing in an important organ, these remedies can be employed with but little expectation of advantage. Respecting my own experience of their effects in these diseases, that is very limited. I have prescribed them more in the progress of recovery from them, with the intention of promoting that process, and at the same time of invigorating the constitution, than to combat their early stages. The manner of prescribing these remedies in the diseases under consideration, has been to give them in small, but sometimes in medium doses, combined with other medicines according to the circumstances of the case.

In *Ovarian Dropsy*.—Having been led to speculate upon the pathology of this malady, from having it subjected to my close observation, and under circumstances of peculiar interest and distress, I resolved to employ other remedies and different modes of treatment from those invariably found hitherto unavailing. In two cases of those which have since fallen under my observation, I have employed the oleum terebinthinæ, or the terebinthina chia, in various proportions, directing them generally so as to be taken into the circulation, and exhibiting them occasionally in a full or maximum dose, so as to produce their full operation upon the alimentary canal. Local depletion and counter-irritants were employed at the same time ; while the full vigour of the nervous energy was promoted by suitable diet and regimen. In one of these cases, the disease has been kept stationary during two years, which was all that could be expected, from the advanced state of the complaint. The other has not been half that period under occasional treatment ; but it commenced at an earlier stage, and the disease is now reduced to a small tumor, perceptible only during some positions of the body.

In *Hydrocephalus.*—1st. In the *acute species* of this disease. In the stage of turgescence, or invasion of the acute hydrocephalus, the oleum terebinthinæ may be employed as an useful auxiliary to other remedies. Nor do I consider that it should be ranked as an auxiliary only, although it may be resorted to in combination with other medicines. No substance, in my opinion, is more calculated to produce a complete derivation of the impetus of the circulation from the brain towards the digestive tube, than the one under consideration. As the stage of turgescence* is merely the progression to the inflammatory state, the mode of treatment and of employing this remedy will differ but little, or at least only as it respects the energy with which it and other remedial agents ought to be entered upon. I have had several opportunities of using this medicine in the Institution for the Diseases of Children, to which I am one of the physicians, and both in the acute and chronic form of this malady. But in a disease which, even in its invasion, threatens the most imminent danger, to trust to the effects of this or any other single remedy, would, in my opinion, be a palpable want of activity in the physician: I have, on that account, employed other means in addition to its use. The submuriate of mercury with digitalis, (the latter especially in the inflammatory stage,) have been prescribed through the day and at night; while local bleedings have been resorted to, and pediluvia, followed by mustard cataplasms to the calves of the legs, have been also enjoined. The manner in which I have employed this oil, has been to give it the first in the morning, in such a combination as to ensure its drastic and derivative effects, and at the same time to prevent it from being ejected by vomiting. That degree of ventricular irritation that gives rise to the sensation of nausea, is not in any degree hurtful in these stages of this disease; but, from the sedative effects that result from it, in every respect the contrary: whereas, if it be increased so as to produce vomiting, augmented impetus of circulation in the head becomes the consequence, which always proves hurtful in the various species of this disease. This maximum dose ought to be continued every morning, especially in the inflammatory stage, until the treatment has effected its removal. In this stage, depletion may be carried to considerable length. Children can bear the loss of blood much better than has been generally supposed. The dread of carrying this mode of cure to an undue extent in the acute diseases of children, has tended greatly to promote a feeling of despair in the mind of the practitioner, when entering upon the treatment of many of their ailments. This sentiment was first combatted in some degree by the late Dr. Clarke; but our progress in pathological research has most materially, and even lately, promoted the advancement of this department of the healing art.

In the inflammatory stage of acute hydrocephalus, the oleum terebinthinæ appears to be one of the best cathartics, and only excelled by the hydrarg. submur.; and, if judiciously employed in conjunction with that remedy and digitalis, with depletion, cold applications to the head, and revulsants to the

* See the admirable works of GOLIS of Vienna, on Acute and Chronic Hydrocephalus; the former has been lately translated by the able hand of Dr. GOOCH.

extremities, the nape of the neck, and between the shoulders, according to circumstances, this species of the malady may be combatted with more certain prospect of success than that hitherto entertained.

In the stage of effusion, this remedy deserves a trial. In one case, in which I believed that state to have existed completely, from the position of the child and disregard of external objects, &c. this medicine was exhibited in small doses through the day, with a maximum quantity on alternate mornings. This plan of treatment was continued for a considerable time, with little variation, unless when the medicine affected the urinary organs; when it was discontinued, and the hydrarg. cum creta, with digitalis and the spirit. æther. nitrici, were substituted for a short time in its place. The child ultimately recovered.

In *chronic Hydrocephalus*, I have employed the oil of turpentine in a number of cases, both directing it in order to produce its diuretic effects, and also as a cathartic. When prescribing it with the former intention, I have sometimes given it along with the digitalis; at other times, continuing its use for a few days only, and then employing mercurials, with digitalis and other diuretics, for an equal period; thus alternating the oil with this class of remedies sufficiently long to ascertain the effect. This mode of resorting to its use is most applicable to that condition of the disease marked by an atonic state of the exhalant and capillary vessels. If further experience shall confirm the good effects which I have considered to have arisen from its use in this and in other dropsical effusions, it may be viewed as owing chiefly to its tonic effect upon these vessels. The convulsions and very constipated state of the bowels which so frequently occur in this species of the disease, are, in my opinion, best combatted by a maximum dose of this medicine. In several cases in which the most active cathartics failed of producing any effect, although exhibited in surprisingly large doses, the oleum terebinthinæ, with half the quantity of the oleum ricini, have produced copious evacuations. Three cases of chronic hydrocephalus, accompanied with complete loss of the functions of the brain, have occurred to me, (two of which were in the Royal Dispensary for Children,) and have been treated in this manner where the patients have so far recovered as to become sensible to the impression of external objects; and, although the disease was evidently only partially removed, yet their parents have considered them sufficiently recovered to leave off the use of medicine. In these and the other cases that have fallen under my observation, and which generally, from their very advanced stage, were particularly hopeless, this substance formed the chief cathartic remedy prescribed, (in addition to its exhibition in small and frequent doses,) and as such it was liberally given. However, under every mode of exhibition, but more especially when given in small doses in this disease, its effects upon the urinary organs, and the degree of distention of the bladder, ought to be examined.

In one case of chronic hydrocephalus in a child of nine months, and in which the head was enormously enlarged, the operation of letting out a portion of the effused fluid by puncture was performed, at my request, by my friend Mr. Dendy. Diuretic remedies were also prescribed. The child died some time after the third repetition of the operation.

In chronic affections of the urinary organs, the terebinthinous remedies may be resorted to with advantage. In all cases of a relaxed state of the vessels in the pelves of the kidneys, or in the mucous membranes covering the urinary passages, remaining after the debilitating effects of acute disease, their use may be attended with great benefit; and with such I have employed both them and the uva ursi; but neither is admissible while any acute symptom remains.

The diuretic and stimulant effects of the oil upon the urinary organs, led Ballonius* to employ it as a remedy in *sterilitas;* and, he says, with advantage in several cases.

I consider both the oil and the terebinthinates very excellent remedies in *fluor albus*, and in diseases of the uterus depending upon a debilitated state of the vessels in that organ and its appendages. They deserve trial also in old *gleets;* and certainly, in that disease, might be expected to exert even a more decided efficacy than the copaiba balsam, which is so similar to them in chemical constitution.

In *Icterus.*—Guyton de Morveau recommended the oleum terebinthinæ, with ether, as a solvent of biliary calculi, and tried many experiments with it among the patients of Dr. Durande. What degree of efficacy it could produce while the calculus remained in the ducts, would be difficult to determine: that it produced any, may be doubted. Darwin and Saunders have both performed experiments, in order to ascertain the solvent power of this substance over biliary calculi when out of the body, which have not been demonstrative of its complete success, when even resorted to in this direct manner. The influence it has been found to possess in the cure of icterus, by Odier† in France, and by Herz‡ and Holst§ in Germany, must, in my opinion, be explained upon a different principle than that of its efficacy in dissolving the calculi obstructing the biliary ducts.

*Against Worms in the Intestinal Canal.*—The oleum terebin-

* *Consultationes Medicæ*, vol. iii. p. 293.

‡ Herz, in Hufeland's *Jour.* b. iii. p. 575. § Holst, in do. b. xx. 2 n. p. 147.

thinæ was recommended as an anthelmintic in the early part of the seventeenth century, by Thomas Bartholinus. Chabert, a French writer, extolled its virtues in that capacity in 1781. Since that period it has been frequently exhibited with this intention, especially against the tæniæ; and certainly no remedy can be resorted to with more certain prospects of success. It is quite unnecessary for me to quote an immense number of cases, detailed in the various Journals and Transactions of Medical Societies, illustrative of this position, as the point is quite established. I have employed it myself against tæniæ in seven cases, in all of which it effected the expulsion of the dead animal: it, therefore, acts in a double capacity in ridding the body of these troublesome inmates, by causing their death and their rejection. I have generally preferred to exhibit this substance against tape-worm in a pure and uncombined state, or nearly so, and frequently upon the surface of some distilled water, with the addition of a drop or two of some fragrant oil. The time best suited to its exhibition is the morning early; it ought to be taken before any thing whatever; nor should food follow its ingestion for at least two hours afterwards. If it does not operate by stool in four or five hours after it has been taken, a dose of the oleum ricini ought to be exhibited. I prefer this mode of prescribing the oleum terebinthinæ in tænia, to that of giving another purgative in combination with it, because, by leaving it to its individual effects for some time, it can the better exert its influence in destroying that animal; while the cathartic follows in sufficient time to prevent any very serious effects from its retention. The quantity of the remedy must be left to the physician prescribing, and its repetition in this manner must be guided by the effects of the first decided dose. My friend Mr. Winstone, of Charterhouse-square, a sagacious and observing practitioner, informed me that, on one occasion of his exhibiting this remedy in tænia, although it was given in a very large dose in combination with another cathartic, and although it produced the expulsion of a very large tænia, it nevertheless affected the urinary organs so as to cause bloody urine. This is, however, but a rare occurrence, and, although highly unpleasant, yet it is productive of no lasting bad effects. This instance may have arisen from an accidental activity of the absorbent system, consequent to a previous abstinence undertaken in order to promote the operation of the remedy.

In concluding this subject, I may add, that in the account of the plague as it occurred at Malta, given by Sir B. Faulkner,* of three cases of recovery from that disease there detailed, two of them arose from the accidental ingestion of an immense dose of this substance combined with camphor.

* *Edinburgh Med. Jour.* vol. x. p. 165.

